# Determining the Reactivity of Selected Biomass Types Considering Their Application in Pyrometallurgical Processes of Metal Production

**DOI:** 10.3390/ma17112691

**Published:** 2024-06-02

**Authors:** Robert Findorak, Lubomir Pikna, Tomasz Matuła, Leszek Blacha, Jerzy Łabaj, Albert Smalcerz, Dorota Babilas

**Affiliations:** 1 Institute of Metallurgy, Faculty of Materials, Metallurgy and Recycling, Technical University of Košice, Letná 1/9, 042 00 Košice, Slovakia; robert.findorak@tuke.sk; 2 Institute of Recycling Technologies, Faculty of Materials, Metallurgy and Recycling, Technical University of Košice, Letná 1/9, 042 00 Košice, Slovakia; lubomir.pikna@tuke.sk; 3 Department of Metallurgy and Recycling, Faculty of Materials Science, Silesian University of Technology, Krasinskiego 8, 40-019 Katowice, Poland; leszek.blacha@polsl.pl; 4 Department of Production Engineering, Faculty of Materials Science, Silesian University of Technology, Krasinskiego 8, 40-019 Katowice, Poland; jerzy.labaj@polsl.pl; 5 Department of Industrial Informatics, Faculty of Materials Science, Silesian University of Technology, Krasinskiego 8, 40-019 Katowice, Poland; albert.smalcerz@polsl.pl; 6 Department of Inorganic Chemistry, Analytical Chemistry and Electrochemistry, Faculty of Chemistry, Silesian University of Technology, Ksiedza Marcina Strzody 9, 44-100 Gliwice, Poland; dorota.babilas@polsl.pl

**Keywords:** reduction, biomass, metallurgical slag, pyrometallurgy

## Abstract

In this paper, results of research on the reactivities of selected biomass types considering their application in pyrometallurgical processes of metal production are presented. Walnut shells, sunflower husk pellets and spent coffee grounds were selected as biomass materials. Their use as potential reducers in the process of metallurgical slag decopperisation is an innovative approach to this subject. The thermogravimetric findings show that all three tested biomass types are classified as highly reactive. The time to reach maximum reactivity ranges from 1.5 to 3 min and, the lowest value is recorded for the sample of spent coffee grounds. The sample hold time of two hours enables copper content reduction to approx. 1 wt% for practically all the reducers tested. A longer duration of liquid slag contact with the reducer results in a decreased copper content in the slag to a value below 1 wt%. Copper concentrations of 0.5 wt% and lower are observed with a hold time of 4 h. The preliminary results indicate that there is great potential for the use of this type of material in non-ferrous metallurgy, which may translate into replacing fossil raw materials and thus introducing the principles of a sustainable process in this case of metal production.

## 1. Introduction

The changes to be introduced in the following years related to a restrictive policy aiming at limiting negative effects on the natural environment will require the implementation of far-reaching alterations in many technologies that involve the so-called carbon footprint. In addition to the coal energy industry, the metallurgical sector is also associated with high levels of carbon compound emissions, particularly in the field of metal production and refining.

One of the methods for reducing carbon dioxide emissions in metallurgical processes may be the application of renewable raw materials (including biomass) as substitutes for conventional fossil fuels. Over several decades, biomass has been primarily applied as a fuel in the energy industry. Regarding metallurgical processes, the use of biomass is a more challenging issue. The requirements for charge materials employed in metallurgical processes are far more demanding than those related to the materials applied in the energy industry. This mainly refers to the chemical composition and the form of biomass delivered to a metallurgical system. which also depends on its strength parameters. Moreover, the use of biomass in a metallurgical system is associated with the release of volatile components at far lower temperatures than those required for the basic chemical reactions involved in metal production, which affects the chemical compositions of emerging gases, their emission to the environment and their effects on the working components of the metallurgical unit.

Pyrometallurgical processes aimed at metal production using both primary and secondary raw materials mostly involve carboniferous resources (coke, coke breeze, anthracite), which serve as energy sources and, indirectly, as reducers. The combustion of some of these materials in metallurgical systems provides the thermal energy necessary to heat the metalliferous charge, triggering a series of endothermic reactions between the charge components. Because of limited oxygen access to the interior part of the metallurgical reactor, coal is also involved in the Boudouard reaction that yields carbon monoxide, being the basic reducer for metal compounds contained in the charge material. One of the parameters that characterise carboniferous materials applied in the pyrometallurgical processes of metal production is their reactivity. The reactivity of a solid reducer is defined as its ability to undergo a chemical reaction with a particular oxidising agent in strictly determined conditions including the temperature, pressure in the system, weight and the specific surface area of the gasifying agent. The selection of the oxidising agent for reactivity tests depends on the intended technological application of the particular fuel.

Reactivity is most frequently measured by the rate [1] determined in the following equation:

*R* = 1/*m*_0_ × *dm*/*dt* (1)

where *m*_0_—the initial weight of the fuel sample;

*m*—the instantaneous weight of the sample;

*dm*/*dt*—the weight loss at 50% carbon conversion.

The conversion degree is determined by the following equation [2]:

*X* = (*m*_0_ − *m*)/*m*_0_ × 100% (2)

Fuel reactivity is a complex parameter influenced by many factors. One of them is the pyrolysis process observed during the initial phase of fuel gasification, where the contents of oxygen and hydrogen in the char decrease while the carbon fraction increases. Another very important factor is the effect of the inorganic fuel component contents on the reactivity of chars (particularly those produced from lignites). The differences in reactivities of chars decrease with an increasing conversion degree. When the conversion degree is 50% and higher, chars generated at various temperatures demonstrate comparable reactivity values [2], which is explained by similar numbers of active sites and the same porosity levels. The duration of pyrolysis itself and the temperature increase are related to a reduced activity of the char [3]. Significant effects on fuel reactivity are demonstrated by the heating rate and the specific surface area (grain sizes) of the char. Higher heating rates and smaller grains promote higher values of char reactivity [4]. The methods of fuel reactivity determination are divided depending on the applied oxidising agent. When carbon dioxide is used (carboxyreactivity), the fuel reacts with CO_2_ in the specified conditions according to the reaction:

C + CO_2_ = 2CO (3)

When oxygen is applied (oxyreactivity), the fuel reacts with oxygen in the specified conditions according to the reaction:

C + O_2_ = CO_2_ (4)

When the process is carried out in a water vapour environment (hydroxyreactivity), the tested fuel reacts with the water vapour in the specified conditions according to the reaction:

C + H_2_O = CO + H_2_ (5)

To determine fuel usefulness in metallurgical processes, research should be conducted with carbon dioxide.

While analysing the application of carboniferous materials in metallurgical processes, it should be noted that their selection in terms of reactivity depends on the type of technological process where they are used. For instance, coke used in the blast furnace processes of crude iron production should demonstrate low reactivity to decrease its specific consumption because of a reduced loss in this part of coal, which, following its transition to carbon monoxide in the Boudouard reaction, is transferred to the process gas. A similar situation is observed in processes of cast iron production, where foundry coke is primarily applied as fuel and ensures the required temperature of the process and proper permeability of the charge materials to guarantee a good level of heat exchange between the gaseous and solid phases. In these technologies, it acts both as fuel and, indirectly, as a reducer of metal compounds contained in the charge. A completely different case refers to metallurgical waste (slag) processing in electric arc furnaces, where the carboniferous material is only a direct or indirect reducer of the charge-contained metal compounds and not a fuel to be combusted to generate the heat necessary for the process.

Determining the potential for biomass application in pyrometallurgical processes requires collecting a series of experimental data. In this paper, the results of a study on the reactivity of selected types of biomass and their application as an alternative to commonly used coke breeze in processes of cupriferous slag reduction in electrical furnaces are presented.

### Cupriferous Slag Reduction

In pyrometallurgical processes, slags are generated as waste material, and their chemical compositions mainly depend on the type of metalliferous raw material processed and the applied technology. Further slag processing or their direct use depends on the contents of valuable metal compounds and the contents of toxic metals, which are environmentally unfriendly and cannot be safely stored. In slags derived from various technological processes of copper production, the fractions of this chemical element widely differ, ranging from 0.5 wt% Cu to 16 wt% Cu [5,6,7,8,9,10,11,12,13,14,15,16,17,18,19]. Moreover, these slags contain heavy metals (e.g., lead, zinc, arsenic, chromium, mercury), and their compounds may pose a major hazard when released into the environment. From an economical point of view, slags containing more than 1% Cu are either applied in decopperisation procedures or turned back to their original processes. Slags with smaller copper contents may be stored or utilised in other ways if they do not contain unacceptable fractions of heavy metals.

Among high-temperature procedures of copper slag processing aimed at valuable metal recovery, reduction processes with coke as a reducer are of the greatest importance. In addition, for several years, there have been attempts to apply alternative reducers, such as various types of biomass or carboniferous waste generated in the coal enrichment processes [20,21,22,23,24,25,26,27,28,29,30,31,32,33].

Metallurgical slags generated during copper production demonstrate complex structures that can be described by applying the ionic slag structure theory. Copper contained in the slag phase can stay there in the form of copper(I) oxide, Cu^+^ cations or metallic inclusions, which mainly results from the oxygen potential depending on the chemical compositions and structures of slags. For silicate slags, the most important components in the slag structure are oxygen bridges. The inclusion of metal oxide (MeO) into the slag leads to bond breakage and the generation of O^2-^ anions, which are neutralised by Me^2+^ cations called modifiers. When there are more silicate–oxygen (SiO_4_^4−^) anions than metal oxides, the slag demonstrates an acidic property. The process of copper removal from the slag is strongly affected by the partial pressure of oxygen, which should be maintained at a possibly low level. With copper elimination from the acidic slag over time, the rate of this process significantly decreases because of the presence of bonds between copper and silicate–oxygen anions. The process of reducing the slag-contained copper oxide with the use of carbon contained in the reducer may take place according to the following reactions:

Cu_2_O + C = 2Cu + CO (6)

2Cu_2_O + C = 4Cu + CO_2_ (7)

Cu_2_O + CO = 2Cu + CO_2_ (8)

An important factor influencing the reduction reaction rate is the mass transfer in the liquid phase. In practice, a forced movement of the liquid phase is beneficial for the coagulation of metal particles and their separation from the slag phase.

When hydrocarbons are released during slag reduction in the metallurgical system, they become an additional medium that causes liquid movement and reduces copper oxides. Such a situation is observed when certain types of biomass are applied as reducers.

Progressing climate changes force us to take extensive actions aimed at reducing the consumption of natural energy resources. This problem particularly affects the metallurgical industry, where we deal with reduction processes using coke or, in general, carbon-bearing raw materials. By introducing the assumptions of the Green Deal, the European Union clearly indicates the departure from or maximum limitation of fossil raw materials used at the industrial scale. One of the possibilities in this area is the use of renewable raw materials, including biomass.

So far, the main experiments related to the use of biomass concerned combustion processes and the production of thermal energy. As a result, in the case of pyrometallurgical processes, there is no extensive research on the course of reduction reactions and the impact of substances contained in biomass on these types of reactions, including cellulose, hydrocellulose and lignin. This knowledge is necessary, on the one hand, to provide an appropriate number of bioreducers to maintain the production volume that ensures the European market demand for metals, and, on the other hand, to learn the mechanism of the reaction, the obtained products and their impact on the elements of the equipment or devices used. Taking into account the diversity of biomass in the form of dendromass (forest wood, wood processing industry, used wood), phytomass (agricultural and garden herbs) and fruit biomass, we collected data on the characteristics of individual groups of renewable raw materials as well as their behaviour in specific conditions, i.e., high temperature, metal-bearing materials will allow us to build a system of suggestions indicating which of these materials will be most suitable for the recovery and production of metals. Pilot research conducted by the authors will allow for the selection of target materials in terms of resources and the efficiency of reduction processes, as well as the interaction between the resulting products and installation elements for application in industrial conditions.

To determine the effects of bioreducer addition on the process of copper-containing slag reduction, experiments of reduction smelting in the presence of walnut shells, sunflower husk pellets and spent coffee grounds were carried out. For the assessment of the efficiency of these reducers, experiments with coke breeze as a standard reducer were also conducted. The authors’ research program involved thermogravimetric analysis to determine biomass reactivity. The tests of metallurgical slag reduction depending on the process duration were taken into account.

## 2. Materials and Methods

Three biomass types were applied in this research as follows: walnut shells, sunflower husk pellet and spent coffee grounds (SCGs). Their chemical compositions are presented in Table 1. Initial tests were also performed on the biomass samples to determine their heat of combustion values (see Table 1). For the tests of slag reduction in the presence of biomass, copper slag was applied containing the following main metals: 10.3 wt% Cu, 2.25 wt% Pb and 11.1 wt% Fe. It should be noted that these metals were present in the slag in the form of oxides [34,35,36,37,38,39,40,41,42].

**Figure 1:**
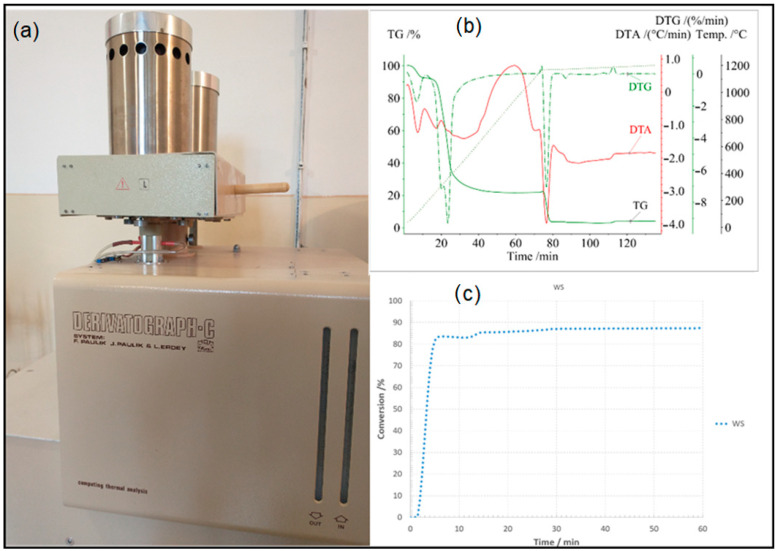
Drawing showing (**a**) Derivatograph-C, (**b**) the TGA curves and (**c**) the conversion curve.

A thermogravimetric analysis (TGA) of the samples was performed in a thermal analyser (Derivatograph-C, MOM Budapest) to study thermal stability and reactivity with CO_2_. The sample was placed in an Al_2_O_3_ crucible. During the first stage, the heating mode was linear with the heating rate of 15 K/min maintained up to 1433 K and subsequently reduced to 0.5 K/min up to 1473 K. The linear heating during the first stage (pyrolysis) was conducted in a N_2_ atmosphere, and the quasi-isothermal second stage (for reactivity) was performed under CO_2_ conditions. The flow rates of the injected gases were constant at the level of 0.05 L/min. Figure 1 illustrates a Derivatograph-C, MOM Budapest, the TGA curves and the conversion curve.

The degree of sample conversion (*X*) was calculated based on the weight loss curve from the onset of its interaction with CO_2_. In this case, the reactivity was determined by the maximum rate (*r_A_*) of weight loss in the monitored interval. Moreover, the assessment factors included the time to reach the maximum rate and the time to reach 50% conversion (t_x50_) during the reaction with CO_2_.

(9) $$r_{A}=\frac{1}{W_{0}}\left(\frac{dW}{dt}\right)$$

(10) $$X=1-\frac{W}{W_{0}}$$

where *W* is the char weight following fixed carbon loss; 

*W*_0_ is the initial char weight; 

(*dW/dt*) is the maximum rate of fixed carbon loss.

**Figure 2:**
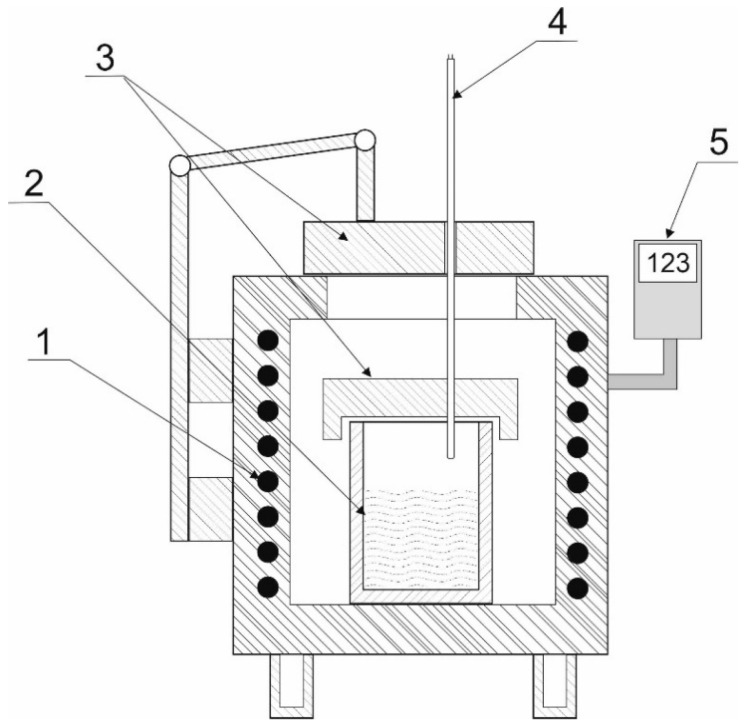
A schematic diagram of the research stand for slag reduction experiments: 1: electric resistance furnace; 2: crucible with the tested sample; 3: ceramic cover; 4: thermocouple; 5: device controlling the furnace operation.

The tests of copper slag reduction were carried out in a Czylok PT 40/1300 (Czylok sp. z o.o, Jastrzebie-Zdroj, Poland) resistance pit furnace (Figure 2). The reduction process was conducted in alundum crucibles at 1573 K. This temperature was determined based on the initial test findings. The slag weight was a fixed parameter of 80 g. The varied parameters were the reduction process duration and the reducer (biomass) addition. Following each experiment, the resulting secondary slag was assessed in a chemical analysis using the ICP-AES method.

The microwave digestion of the slag samples was carried out using a MARS laboratory system (CEM Corp., Matthews, NC, USA). Teflon digestion vessels were used in the experiment. This type of vessel allowed the experiment to be performed by applying a wider range of acid ratios and temperatures. During the experiments, several parameters of the microwave digestion were varied, namely, the acid concentration, the temperature, the hold time and the hold temperature. The volumes of HNO_3_ and HCl were 10 mL and 3 mL, respectively; the temperature was 200 °C, the output power was 900 W and the hold time was 15 min. 

The concentrations of the elements in the slags after microwave digestion were determined using the inductively coupled plasma atomic emission spectrometry (ICP-AES) method. A Varian 710-ES spectrometer (Agilent, Mulgrave, Australia), equipped with a glass SeaSpray nebulizer (Glass Expansion, Port Melbourne, Australia), and a double-pass glass cyclonic spray chamber were applied. The concentrations of the selected elements were determined using the calibration curve method within the range of its linearity. The standards were prepared by diluting the standard solution of the selected elements (1000 mg/L, Certipur, Merck, Darmstadt). A linear model of the calibration curve was assumed, with a minimum correlation coefficient of 0.999. All the results are presented as average values of three replicate measurements.

## 3. Results

### 3.1. Biomass Reactivity

**Figure 3:**
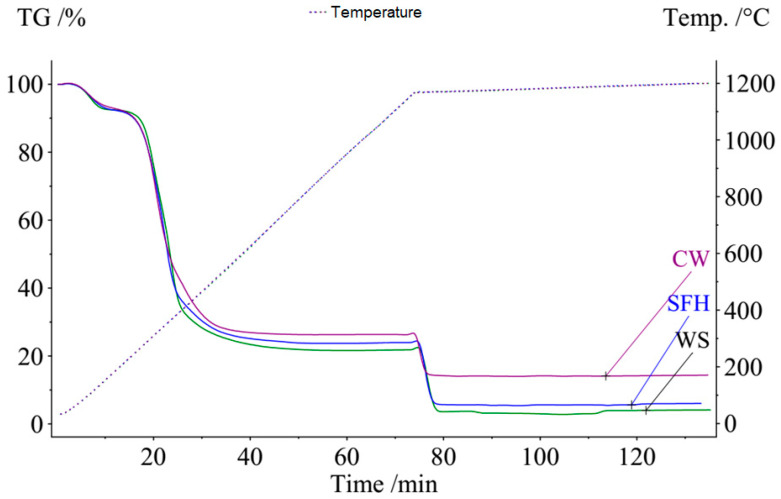
TG curves of the samples yielded during the linear heating in the inert atmosphere and the isothermal heating in CO_2_.

In Figure 3, TG curves derived in the reactivity tests of the coffee ground (SCG), sunflower husk (SFH) and walnut shell (WS) samples are presented.

The calculated parameters from the TG analysis (Table 2) prove that the SFH reactivity is the highest among the three samples. In general, all three samples are classified as highly reactive, but we can see certain differences in the other monitored parameters. The time to reach the maximum reactivity ranged from 1.5 to 3 min, and the lowest value was recorded for the sample of spent coffee grounds. The time to reach 50% conversion in the CO_2_ atmosphere was more favourable for the SFH pellets but with small differences. A significant difference was observed in the inert conditions between the weight loss values because of the volatile components and possible moisture. The SFH and WS samples demonstrated significant weight loss (from 73% to 78%). The remaining part reacted relatively quickly with CO_2_ (as shown in the figure).

**Figure 4:**
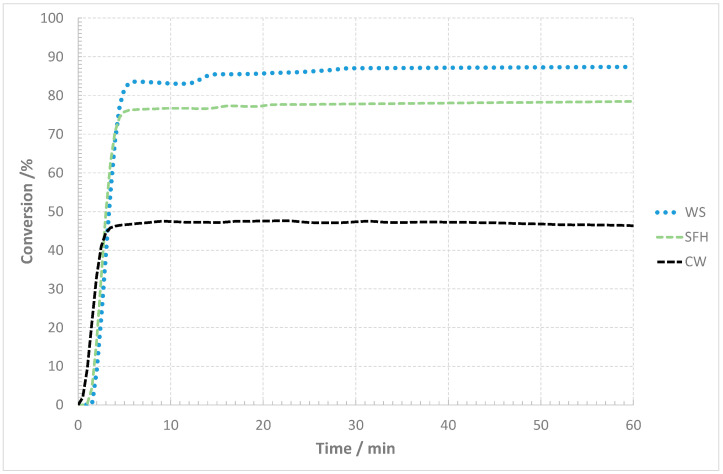
Conversion progress during the reactivity test.

**Figure 5:**
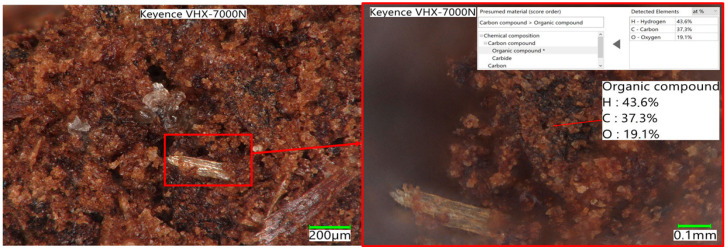
Spent coffee grounds.

**Figure 6:**
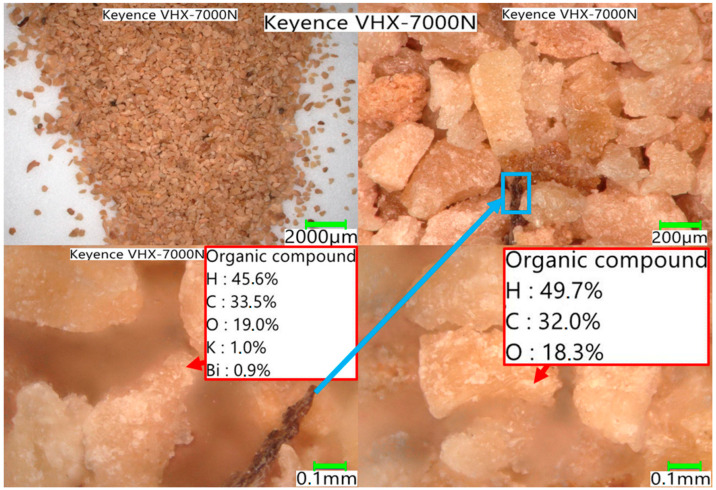
Walnut shells.

**Figure 7:**
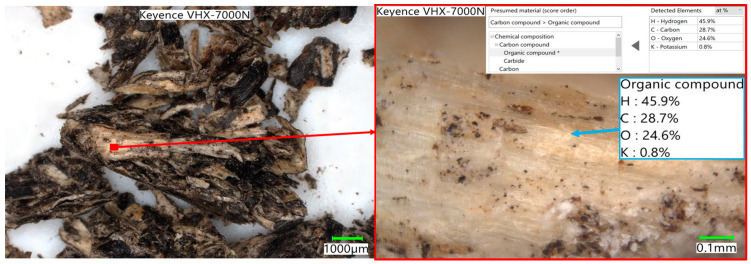
Sunflower husk pellets.

The degree of conversion (Figure 4) also shows significant differences in the maximum values reached as well as in the rate change itself. The 50% conversion within the reactivity test indicates a high conversion rate, but in the case of the spent coffee ground residue, a maximum of 48% was reached. In Figure 5, Figure 6 and Figure 7, the macrostructures of the samples observed using a 3D microscope (Keyence Corporation, Mechelen, Belgium) with the corresponding point analysis are presented.

### 3.2. Reduction Melting of Slags

In Table 3, the results of copper slag reduction melting processes with the selected biomass types used as reducers are presented. In addition to the basic test parameters, the table contains the weight values for the smelted metal and the secondary slag following the reduction process as well as the values of the relative slag decopperisation degree *S_Cu_* (estimated based on the experimental results). To determine the *S_Cu_* values, the following equation was applied:

*S_Cu_* = (*C*^0^*_Cu_* − *C^t^_Cu_*)/*C*^0^*_Cu_* × 100% (11)

where *C^0^_Cu_* and *C^k^_Cu_* are the initial fraction of copper in the slag and the content of copper in the slag after time t (wt%), respectively.

**Figure 8:**
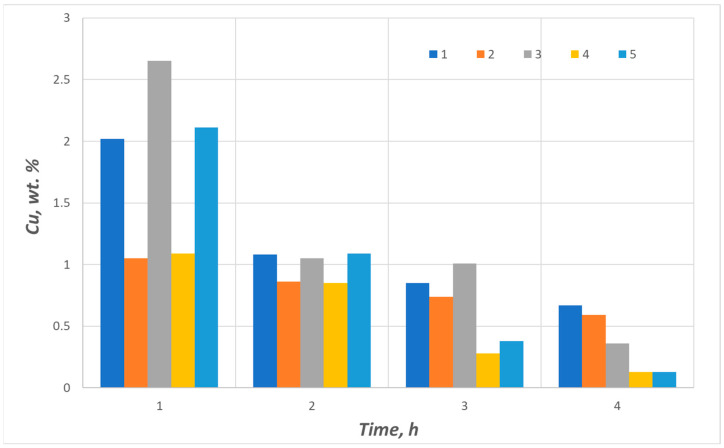
Results of the copper slag reduction tests using the following reducers: 1—coke breeze, 2—flotation concentrate, 3—spent coffee grounds, 4—sunflower husk pellets and 5—walnut shells.

Graphical interpretations of the results are shown in Figure 8. It also presents the results of the copper slag reduction tests in the presence of coke breeze as a conventional reducer in industrial conditions and the fine-grained carboniferous waste generated during the coal enrichment process (coal flotation concentrate).

The results of the laboratory tests of copper slag reduction with biomass as a reducer were compared to the results of tests using breeze coke and flotation concentrate. The test duration ranged from 1 h to 4 h to verify the efficacy of the reducer addition. The hold time of two hours enables copper content reduction to approx. 1 wt% for practically all reducers tested. A longer duration of liquid slag contact with the reducer results in a decrease in the copper content in the slag to values below 1 wt%. Copper concentrations of 0.5 wt% and lower are observed with a hold time of 4 h. In the case of the reduction tests in the presence of sunflower husk pellets and walnut shells, the copper fraction reaches the value of 0.13 wt% Cu at three hours, which is important for practical reasons as the slag containing less than 0.5 wt% complies with the requirements of safe storage or its application in other economic sectors, e.g., the road construction industry. Moreover, considering the process duration, its reduction may result in significantly lower energy consumption and, in the Polish energy sector, limitation of the carbon footprint.

The research findings require further verification at a larger scale to determine the effects of the resulting products on the working components of the metallurgical system and to identify emerging gaseous products. Furthermore, larger-scale research findings, following the identification of the biomass resources and their sizes, will determine the economic value of the selected variant or the material applied in the metallurgical process.

The research carried out on the possibility of using biomass materials in the form of sunflower husk pellets, SCGs and walnut shells in metal reduction processes indicates the possibility of replacing standard reducers in the form of coke or coal flotoconcentrate. With the equivalent carbon content in biomass raw materials corresponding to a raw material such as coke, in the case of copper slag reduction under the applied reduction process test conditions, after four hours using sunflower husk pellets or walnut shells as a reducer, a 4–5 times lower final copper content in the slag was obtained. In the case of SCGs, the copper content in the final slag was 1.5 to 2 times lower. Based on the analysis enabling the determination of the share of volatile parts in biomaterials, it can be concluded that it significantly influences the reduction process, which shows the potential of this type of reducer.

## 4. Conclusions

In the following years, the metal production industry will undergo significant alterations related to the ‘Fit for 55’ and ‘Fit for 90’ packages in the EU. The present coal-based technologies will have to be changed as CO_2_ emission fees will considerably increase production costs and negatively affect their competitiveness and profitability. Biomaterials that may be used in metallurgical processes will improve their economics; however, the technological usefulness of these resources should be determined based on extensive research and technical testing.

The results of the laboratory tests performed in this study concerning the application of waste biomaterials, i.e., coffee spent grounds, sunflower husk pellets and walnut shells, led to the following conclusions:

1. All three analysed samples are classified as highly reactive, but we can see certain differences in other monitored parameters.
2. The time to reach maximum reactivity ranged from 1.5 to 3 min, and the lowest value was recorded for the sample of spent coffee grounds.
3. While using the waste biomaterials (coffee spent grounds, sunflower husk pellets and walnut shells), the potential for slag decopperisation at levels comparable to those for breeze coke (a conventional reducer) was confirmed.
4. The application of reducer addition, such as sunflower husk pellets or walnut shells, ensures copper content reduction at three hours to values lower than 0.5 wt%, which means that the applied waste slag can be safely stored or used as a raw material in other economic sectors.
5. The industrial application of biomaterials requires a series of larger-scale tests to determine the effects of volatile matter on the atmosphere and its corrosive effects on the working components of the metallurgical system as well as on the emission of gaseous pollutants generated during the process.

## Figures and Tables

**Table 1:** Chemical compositions of tested biomass samples.

Reducer	Chemical Composition of the Reducer, wt%					Heat of Combustion
	C	H	O	N	S	kJ/kg
Walnut shells	46.30	6.59	41.66	0.50	-	17.35
SCGs	47.20	6.17	50.00	3.11	-	21.09
Sunflower husk pellet	52.08	6.06	37.94	0.75	0.09	20.49

**Table 2:** Parameters of reactivity for the tested biomass samples.

Sample	r_A_/min^−1^	t_rA_/min	t_X50_/min	Δm_INERT_/%	m_FINAL_/%
WS	0.366979773	3	3.86	78.01	2.78
Pellet SFH	0.414915945	2.5	3.02	75.88	5.20
Spent coffee grounds	0.18344614	1.5	4.60 * (46.6)	3.25	14.35

* time for the 46.6% conversion degree.

**Table 3:** The results of copper slag reduction melting processes with the selected biomass types used as reducers.

Sample No	Reducer	Reducer Weight, g	Time, h	Metal Weight, g	Secondary Slag Weight, g	Cu Content, wt%
1	coke breeze	10.12	1	2.05	76.2	2.02
2			2	3.5	73.7	1.08
3			3	6.7	69.89	0.85
4			4	8.41	67.16	0.67
5	flotation concentrate	11.72	1	10.45	70.66	1.05
6			2	10.5	67.96	0.86
7			3	11.99	64.88	0.74
8			4	11.53	66.02	0.59
9	coffee	18.28	1	7.95	68.83	2.65
10			2	12.7	62.69	1.05
11			3	7.98	69.51	1.01
12			4	12	64.54	0.36
13	pellet	18.28	1	11.03	66.08	1.09
14			2	12.6	62.04	0.85
15			3	7.56	70	0.28
16			4	12.26	62.05	0.13
17	walnut	18.28	1	9.93	66.36	2.11
18			2	8.97	67.06	1.09
19			3	12.65	62.83	0.38
20			4	12.26	62.05	0.13

## Data Availability

Data is contained within the article.
